# Berberine protects against gefitinib-induced liver injury by inhibiting the HMGB1/TLR4/NF-κB pathway

**DOI:** 10.3389/fphar.2025.1645634

**Published:** 2025-08-26

**Authors:** Qiongyin Zhang, Na Li, Xue Ma, Yue Qiu, Chao Li, Ya Chen

**Affiliations:** ^1^ Department of Pharmacy, Chongqing University Cancer Hospital, Chongqing, China; ^2^ Department of Pharmacy, Sichuan Clinical Research Center for Cancer, Sichuan Cancer Hospital & Institute, Sichuan Cancer Center, Affiliated Cancer Hospital of the University of Electronic Science and Technology of China, Chengdu, China

**Keywords:** gefitinib, berberine, drug-induced liver injury (DILI), HMGB1/TLR4/NF-κB, hepatoprotection

## Abstract

**Background:**

Gefitinib (GEF), a first-generation epidermal growth factor receptor (EGFR) tyrosine kinase inhibitor (TKI) for non-small cell lung cancer (NSCLC), is frequently associated with drug-induced liver injury (DILI), thereby limiting its clinical application. This study aimed to evaluate the hepatoprotective effects of berberine (BBR) and explore the underlying mechanisms.

**Methods:**

*In vitro*, human hepatocyte lines (THLE-2 and THLE-3) were exposed to GEF alone or in combination with HMGB1 siRNA, a TLR4 inhibitor, an NF-κB inhibitor, or varying concentrations of BBR to assess hepatotoxicity and the involvement of the HMGB1/TLR4/NF-κB pathway. *In vivo*, Sprague-Dawley (SD) rats were treated with GEF with or without different doses of BBR for 21 days. Liver injury and inflammatory responses were assessed, and pathway alterations were evaluated at both transcriptional and protein levels.

**Results:**

GEF activated the HMGB1/TLR4/NF-κB pathway *in vitro*, increasing the levels of p-NF-κB p65, ALT, AST, and pro-inflammatory cytokines (INF-α, IL-1β and IL-6). BBR inhibited these effects in a concentration-dependent manner by suppressing pathway activation, reducing hepatotoxicity, and inhibiting HMGB1 nuclear-to-cytoplasmic translocation. *In vivo*, GEF induced weight loss, an increased liver-to-body weight ratio, elevated serum transaminases and pro-inflammatory cytokines, and histopathological liver injury, all of which were dose-dependently ameliorated by BBR co-administration. Moreover, BBR significantly downregulated the expression of HMGB1, TLR4, and NF-κB at both mRNA and protein levels in liver tissues.

**Conclusion:**

GEF-induced liver injury is mediated by HMGB1-driven inflammation via the TLR4/NF-κB pathway. BBR provides dose-dependent hepatoprotection by targeting this pathway, suggesting a potential strategy to protect against GEF-induced liver injury among NSCLC patients.

## 1 Introduction

Targeted therapies have revolutionized cancer treatment, offering significant improvements in survival rates and the quality of life for patients. Gefitinib (GEF), a first-generation epidermal growth factor receptor (EGFR) tyrosine kinase inhibitor (TKI), has become a widely used small-molecule targeted drug in the treatment of non-small cell lung cancer (NSCLC). Despite its efficacy, GEF therapy is often complicated by adverse effects, with drug-induced liver injury (DILI) being one of the most common and concerning complications (Zhou et al., 2024). GEF-induced hepatotoxicity spans silent elevations in serum aminotransferases to life-threatening liver injury, often necessitating dose reduction or treatment cessation. Specifically, GEF carries a markedly higher risk of serum aminotransferase elevation than other EGFR-TKIs (Ding et al., 2017; Wu et al., 2021), with incidence rates ranging from 39% to 70.1% (Shi et al., 2025; Mitsudomi et al., 2010). Furthermore, nearly 8%–35.1% of patients may require discontinuation of GEF due to severe hepatotoxicity (Common Terminology Criteria for Adverse Events ≥ Grade 3) (Shi et al., 2025; Mitsudomi et al., 2010; Mok et al., 2009). Such adverse events not only limit the clinical utility of GEF but also compromise patient compliance and therapeutic outcomes, highlighting the urgent need for effective and clinically applicable hepatoprotective strategies.

Although the precise mechanism of gefitinib-induced liver injury remains to be fully elucidated, accumulating evidence suggests that high mobility group box 1 (HMGB1) is critically involved in its pathogenesis. Gefitinib has been reported to promote the release of HMGB1 from cells, thereby initiating a sterile inflammatory response (Noguchi et al., 2021). In addition, Zhang et al. (2015) reported that glycyrrhizic acid, an HMGB1 inhibitor, can mitigate gefitinib-induced cellular effects by inhibiting HMGB1 release. These findings collectively highlight HMGB1 as a key mediator of gefitinib-induced inflammation. HMGB1 is known to play an essential role in the development of both acute and chronic liver diseases (Yang and Tonnesseen, 2019). Under conditions of cellular stress or injury, HMGB1 translocates from the nucleus to the cytoplasm or is released extracellularly, where it functions as a damage-associated molecular pattern (DAMP) to trigger inflammatory responses (Khambu et al., 2019). Once released, HMGB1 interacts with pattern recognition receptors such as the receptor for advanced glycation end products (RAGE) and Toll-like receptors (TLRs), particularly TLR4, thereby activating downstream signaling pathways including nuclear factor κB (NF-κB) (Ni et al., 2021). Activation of the HMGB1/TLR4/NF-κB signaling cascade leads to the production and secretion of pro-inflammatory cytokines, ultimately exacerbating liver injury. Several studies have shown that pharmacological modulation of this pathway using compounds such as isochlorogenic acid A (Liu et al., 2020), kaempferol (Du et al., 2020), and paeoniflorin (Xie et al., 2018) confers hepatoprotective effects. These findings suggest that targeting the HMGB1/TLR4/NF-κB signaling axis may represent a promising therapeutic strategy for mitigating gefitinib-induced hepatotoxicity.

Berberine (BBR), a natural isoquinoline alkaloid traditionally used to treat gastrointestinal infections, has garnered increasing attention for its hepatoprotective properties (Ionita-Radu et al., 2024). Increasing evidence indicates that BBR modulates multiple signaling pathways involved in the pathogenesis of liver diseases, particularly offering protection against DILI through various mechanisms (Bansod et al., 2021; Zhou et al., 2021). Notably, studies have shown that BBR can modulate HMGB1-related pathways, reducing inflammation in various models of liver injury. Berberine pretreatment has been shown to lower HMGB1, p-p65, and cleaved caspase-1 levels, thereby alleviating acetaminophen-induced liver damage (Zhao et al., 2018). It also inhibits the HMGB1/NF-κB pathway, reducing cytokine release and improving outcomes in subarachnoid hemorrhage (Zhang et al., 2020). Additionally, berberine inhibits HMGB1 and NF-κB protein translocation, providing protection against ischemia-reperfusion injury (Zhu et al., 2018). Other studies report that BBR mitigates myocardial ischemia and reduces HMGB1 release in LPS-stimulated macrophages (Zhang et al., 2014; Lee et al., 2013).

These findings indicate that BBR may exert hepatoprotective effects by modulating the HMGB1/TLR4/NF-κB pathway. This study therefore examines whether BBR can safeguard against GEF-induced hepatotoxicity by targeting the HMGB1/TLR4/NF-κB pathway, potentially establishing it as an adjunct in EGFR-TKI therapy.

## 2 Materials and methods

### 2.1 Materials

BBR were purchased from Coolaber Reagent (Beijing, China). GEF was purchased from MACKLIN Reagent (Shanghai, China). TUNEL Apoptosis Detection Kit (FITC) was purchased from YEASEN (Shanghai, China), and immunostaining permeabilization solution (Triton X-100) were purchased from Beyotime Reagent (Shanghai, China). Human and rat tumor necrosis factor-alpha (TNF-α), interleukin-1 beta (IL-1β), and interleukin-6 (IL-6) enzyme-linked immunosorbent assay (ELISA) kits were purchased from Thermo Fisher Scientific (Massachusetts, United States) and Elabscience Biotechnology (Wuhan, China), respectively. The primers for RT-qPCR were synthesized by Qingke Biotechnology (TSINGKE, Shenzhen, China). The specific inhibitors for NF-κB (SC75741) and TLR4 (TLR4-IN-C34) were purchased from Selleck. cn (Shanghai, China). GAPDH antibody, rabbit anti-HMGB1 antibody, rabbit anti-TLR-4 antibody were purchased from Proteintech (Wuhan, China). Rabbit anti-NF-κB (p65) and rabbit anti-p-NF-κB [p-p65(Ser536)] antibody were purchased from Affinity Biosciences (Jiangsu, China). HRP-Goat Anti-Rabbit IgG (H + L) and Goat-Anti-Rabbit IgG/Alexa Fluor 488 was purchased from ZSGB. BIO (Beijing, China). (Specific antibody information is provided in Supplementary Table S1).

### 2.2 Cell culture

The THLE-2 cells were obtained from Xiamen Immocell Biotechnology Co.,Ltd. (Xiamen, China), and the THLE-3 cells were provided by Professor Wang Ling’s team (Sichuan University, Chengdu, China). All cells were cultured in basic DMEM (Thermo Fisher Scientific, American) supplemented with 10% fetal bovine serum, 100 μg/mL of penicillin/streptomycin mixture at 37 °C in a 5% of CO_2_ atmosphere.

Based on preliminary CCK-8 cytotoxicity assays and referenced literature reports (Chen et al., 2019; Xu et al., 2018), we selected appropriate drug concentrations for the study: gefitinib (10 μM) for hepatotoxicity induction and berberine (5, 10, 20 μM) for evaluating protection against gefitinib-induced injury.

### 2.3 Animals and drug treatment

Male SD rats (4–5 weeks old, 100–150 g) were purchased from Chongqing Tengxin biotechnology co., LTD (Chongqing, China). The animals were kept in polycarbonate cages (6/cage) under 12 on/off light cycles, feed and water *ad libitum*. All animal experiments were conducted in accordance with the principles outlined in the Guide for the Care and Use of Laboratory Animals by the National Institutes of Health and were approved by the Animal Ethics Committee of Chongqing University Cancer Hospital (Chongqing, China), in compliance with the ARRIVE guidelines.

Following a 7-day quarantine period, rats were randomly assigned to one of six groups (n = 8 per group): (1) Blank control (no treatment); (2) Vehicle control (0.5% sodium carboxymethyl cellulose, CMC-Na with 0.04% Tween 80); (3) GEF (100 mg/kg); (4) GEF + low-dose berberine (BBR-L, 25 mg/kg); (5) GEF + medium-dose berberine (BBR-M, 50 mg/kg); and (6) GEF + high-dose berberine (BBR-H, 100 mg/kg). The gefitinib-induced rat liver injury model and berberine co-administration dosing regimens were based on results from preliminary *in vivo* experiments and relevant published studies (Chen et al., 2021; Mehrzadi et al., 2018; Mahmoud et al., 2017). Due to limited solubility, GEF and BBR were administered orally by gavage as suspensions in 0.5% CMC-Na with 0.04% Tween 80. The GEF group received GEF and the CMC-Na/Tween 80 vehicle. In the GEF + BBR groups, BBR was administered via gavage, followed 4 h later by gefitinib (100 mg/kg). All treatments were administered daily for 21 days. Body weight was measured daily throughout the treatment period. At the conclusion of the 21-day treatment, rats were anesthetized via pentobarbital injection, and blood samples were collected via cardiac puncture. Livers were then excised, weighed, and snap-frozen in liquid nitrogen before being stored at −80 °C until further analysis. A schematic representation of the experimental design is provided in Figure 5A.

### 2.4 Estimation of ALT and AST levels

The alanine aminotransferase (ALT) and aspartate aminotransferase (AST) levels in rat serum were determined by Scientist BIOTECHNOLOGY (Sichuan, China). For cell supernatants, ALT and AST levels were measured using Alanine Aminotransferase (ALT/GPT) Activity Assay Kit and Aspartate Aminotransferase (AST/GOT) Activity Assay Kit which were purchased from Elabscience Biotechnology (Wuhan, China).

### 2.5 Histological examination

The liver tissues were fixed in neutral formalin solution for at least 48 h, then embedded in paraffin, and sectioned at a thickness of 4 μm for histopathological evaluation. The tissue sections were stained with hematoxylin and eosin (HE), and the histopathological changes were observed under a light microscope (Precipoint-M8 Digital Scanning Microscopic Imaging System, Germany, 200×).

### 2.6 siRNA interference

The THLE-2 and THLE-3 cells were transfected with 50 nM of HMGB1-targeting siRNAs (5′-GGA​GAG​AUG​UGG​AAU​AAC​ATT-3′, 5′-UGU​UAU​UCC​ACA​UCU​CUC​CTT -3′) (HanBio, Shanghai, China) using Lipofectamine™ 3000 (Thermo Fisher Scientific, United States) following the manufacturer’s instructions. Cells were collected for subsequent analysis 48 h post-transfection.

### 2.7 RT-qPCR analysis

Total RNA was isolated from rat liver and THLE-2/THLE-3 cells using TRNzol reagent (TIANGEN, Beijing, China), and cDNA was synthesized using the HifairⅢfirst Strand cDNA Synthesis SuperMix for qPCR (gDNA digester plus) (YEASEN, Shanghai, China). RT-qPCR was performed using Hieff^®^ qPCR SYBR Green Master Mix (No Rox) (YEASEN, Shanghai, China) and primers specific for human/rat HMGB1/Hmgb1, TLR4/Tlr4, and GAPDH/Gapdh on the QX-Real-time PCR System (JLM, Sichuan, China). GAPDH/Gapdh served as the internal control, and relative expression was calculated using 2^−ΔΔCt^ method. The primer sequences employed throughout this study are listed in Table 1.

**TABLE 1:** Oligonucleotide primers for gene expression analysis by RT-qPCR.

Oligoname	Sequence (5′-3′)
human-HMGB1	F-5′-TATGGCAAAAGCGGACAAGG-3′ R-5′-CTTCGCAACATCACCAATGGA-3′
human-TLR4	F-5′-AGACCTGTCCCTGAACCCTAT-3′ R-5′-CGATGGACTTCTAAACCAGCCA-3′
human-GAPDH	F-5′-GTCAACGGATTTGGTCGTATTG -3′ R-5′-TGTAGTTGAGGTCAATGAAGGG-3′
rat-Hmgb1	F-5′-TATGGCAAAGGCTGACAAGG-3′ R-5′-TTTCTTCGCAACATCACCAA-3′
rat-Tlr4	F-5′-CCAGAGCCGTTGGTGTATCT-3′ R-5′-CAGAGCATTGTCCTCCCACT-3′
rat-Gapdh	F-5′-GGCACAGTCAAGGCTGAGAATG-3′ R-5′-ATGGTGGTGAAGACGCCAGTA-3′

### 2.8 Western blot analysis

Total proteins were extracted from lysate of rat liver tissues and THLE-2/THLE-3 cells using RIPA buffer supplemented with PMSF. An appropriate amount of protein was loaded onto a 10% SDS-PAGE gel. Subsequently, the proteins were transferred to PVDF membranes. The membranes were then incubated with primary antibodies at 4 °C overnight. After incubation with HRP-Goat Anti-Rabbit IgG (H + L) secondary antibody, protein bands were visualized using an ECL detection kit (YEASEN, Shanghai, China) and imaged with a ChemiDoc MP imaging system (Bio-Rad, United States). The intensity of the bands was quantified by means of ImageJ software.

### 2.9 INF-α, IL-1β and IL-6 analysis

The levels of TNF-α, IL-1β, and IL-6 in the cell supernatants and rat serum were quantified using commercially available ELISA kits, according to the manufacturer’s instructions.

### 2.10 TUNEL assay

Cell apoptosis was detected using the TUNEL Apoptosis Detection Kit (FITC) (YEASEN, Shanghai, China). Cells were seeded on coverslips in 24-well plates and cultured for 48 h at 37 °C in a 5% CO_2_ incubator. After fixing with 4% paraformaldehyde for 30 min and permeabilizing with Triton X-100, the cells were incubated with 1×Equilibration buffer for 20 min at room temperature. Next, TdT incubation solution was applied for 60 min at 37 °C in the dark. Following PBS washes, DAPI staining was performed for 2 min, and anti-fluorescence quencher (Beyotime, Shanghai, China) was added. TUNEL-positive cells were imaged using an Olympus IX73 microscope (400×).

### 2.11 Immunofluorescence detection of HMGB1 translocation

HMGB1 nuclear-to-cytoplasmic translocation were assessed by immunofluorescence in THLE-2 and THLE-3 cells. Cells were seeded on coverslips in 24-well plates and cultured for 48 h at 37 °C in a 5% CO_2_ incubator, then fixed with 4% paraformaldehyde for 30 min, permeabilized with Triton X-100, and blocked with 1% BSA. After overnight incubation with primary HMGB1 antibody (1:200, Proteintech, 10829-1-AP) at 4 °C, slices were treated with Alexa Fluor 488-conjugated secondary antibody (1:50, ZSGB-BIO, ZF-0511). HMGB1 localization was imaged using an Olympus IX73 microscope (200×, 400×).

### 2.12 Statistical analysis

Data analysis in this study was performed using GraphPad (version 9.5) and Origin (version 2021), and results are presented as mean ± standard deviation. The differences in means between the groups were assessed by Student’s t-test based on the data analysis. Statistical significance was established when the p-value was less than 0.05.

## 3 Results

### 3.1 GEF induces HMGB1, TLR4, and NF-κB expression and elevates ALT, AST, and inflammatory cytokines, while inhibitors reduce hepatocyte injury

**FIGURE 1:**
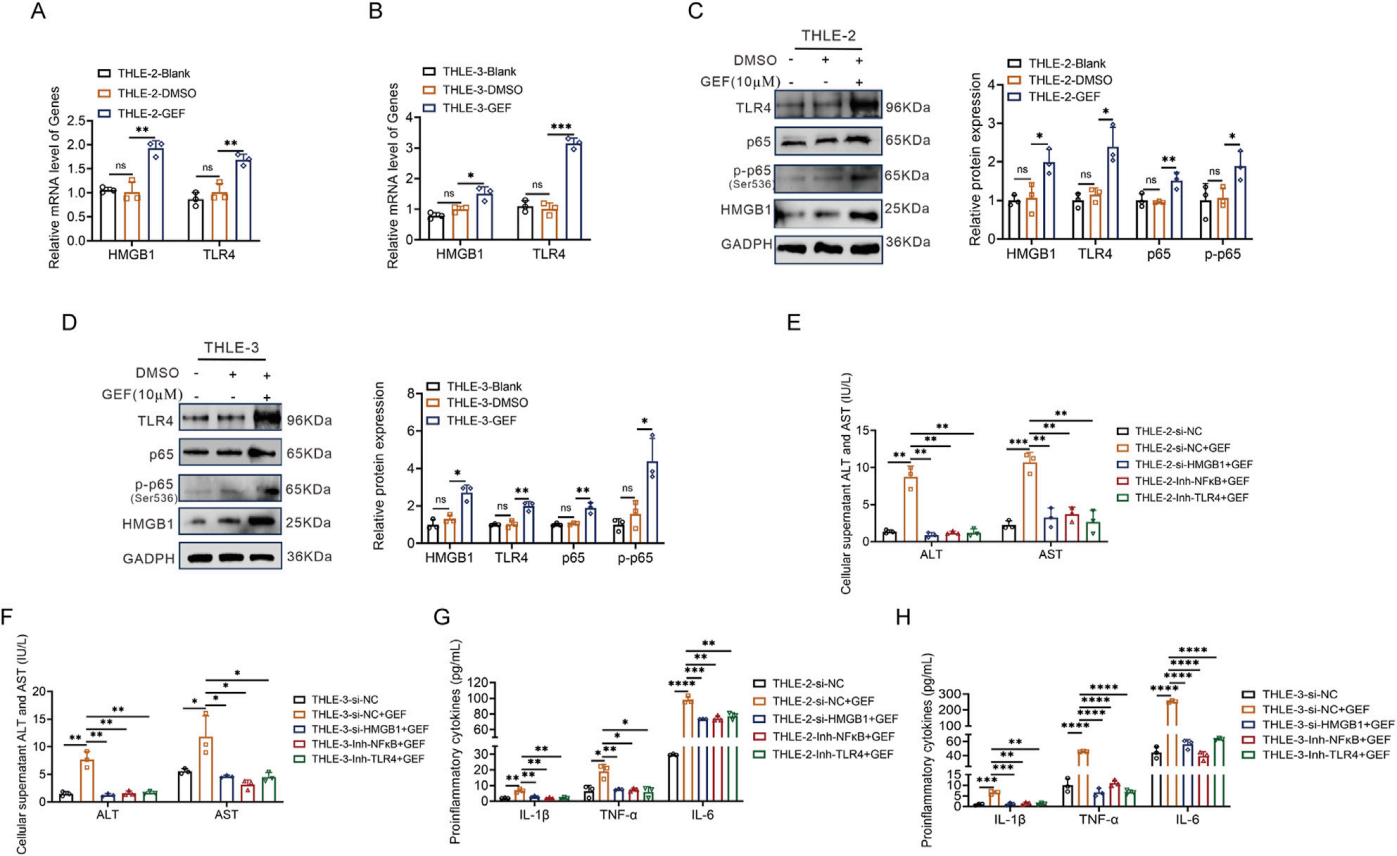
Gefitinib (GEF) induced upregulation of HMGB1, TLR4, and NF-κB expression, and promotes hepatocyte injury in THLE-2 and THLE-3 cells (n = 3). **(A,B)** mRNA expression of HMGB1 and TLR4. **(C,D)** Protein levels of HMGB1, TLR4, and NF-κB (p65 and p-p65). **(E,F)** ALT and AST levels in the cell supernatant. **(G,H)** Inflammatory cytokine levels (TNF-α, IL-1β, and IL-6) in the cell supernatant. Cells were treated with GEF (10 μM) for 48 h or co-treated with HMGB1 siRNA, TLR4 inhibitor (TLR4-IN-C34, 20 μM) or NF-κB inhibitor (SC75741, 1 μM). ns, no significance. *P < 0.05, **P < 0.01, ***P < 0.001, ****P < 0.0001. P-value < 0.05 was considered statistically significant.

To initially investigate the mechanism of GEF-induced hepatotoxicity and the role of the HMGB1/TLR4/NF-κB pathway, THLE-2 and THLE-3 cells were exposed to GEF (10 μM) for 48 h. The mRNA expression of HMGB1 and TLR4 was measured by quantitative PCR (qPCR), and Western blot was used to assess the protein levels of HMGB1, TLR4, and NF-κB (including p65 and its phosphorylated form, p-p65). GEF treatment resulted in a significant upregulation of HMGB1 and TLR4 mRNA expression (Figures 1A,B). Additionally, the protein levels of HMGB1, TLR4, and NF-κB (p65 and p-p65) were markedly elevated (Figures 1C,D). Notably, the increase in p-p65 protein expression exceeded that of p65, indicating significant activation of NF-κB. These findings indicate that GEF activates these key molecular components.

Subsequently, THLE-2 and THLE-3 cells were co-treated with GEF and either HMGB1 small interfering RNA (siRNA), a TLR4 inhibitor (TLR4-IN-C34), or an NF-κB inhibitor (SC75741) for 48 h. The levels of ALT, AST, and inflammatory cytokines (TNF-α, IL-1β, and IL-6) in the cell supernatant were measured by ELISA. The results revealed that each co-treatment significantly reduced the elevation of ALT, AST, and pro-inflammatory cytokines induced by GEF (Figures 1E–H). These findings indicate that inhibition of HMGB1, TLR4, or NF-κB mitigates GEF-induced hepatocyte injury and inflammatory responses.

### 3.2 HMGB1 is critical for GEF-induced activation of the TLR4/NF-κB pathway and hepatotoxicity

**FIGURE 2:**
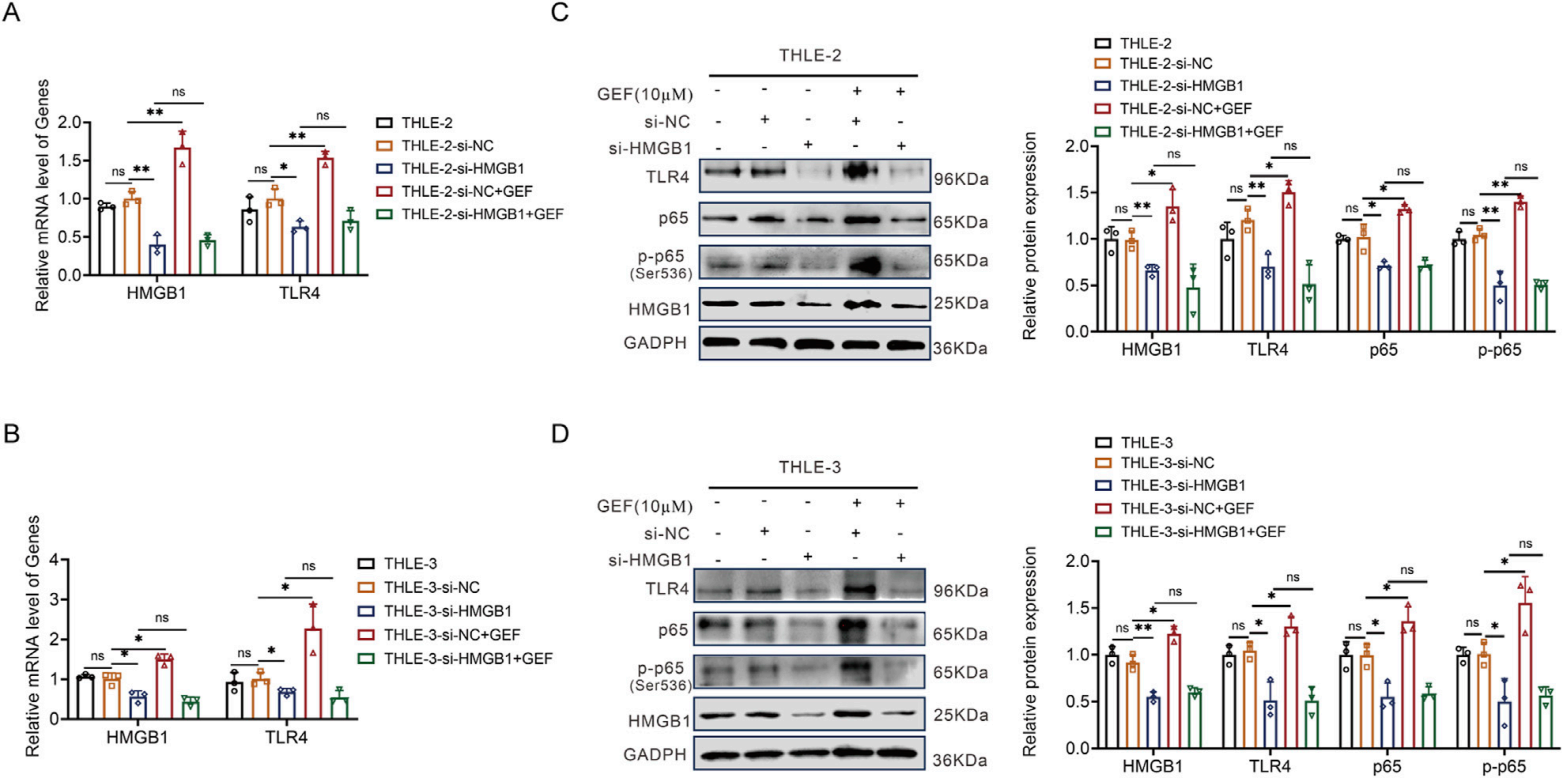
HMGB1 siRNA reversed the induction of TLR4 and NF-κB by gefitinib (GEF) in THLE-2 and THLE-3 cells (n = 3). **(A,B)** mRNA expression of HMGB1 and TLR4. **(C,D)** Protein levels of HMGB1, TLR4, and NF-κB (p65 and p-p65). Cells were pretreated with HMGB1 siRNA, followed by GEF (10 μM) treatment for 48 h. ns, no significance. *P < 0.05, **P < 0.01, ***P < 0.001, ****P < 0.0001. P-value < 0.05 was considered statistically significant.

To clarify the upstream-downstream regulatory relationships among HMGB1, TLR4, and NF-κB in GEF-induced liver injury, THLE-2 and THLE-3 cells were pretreated with HMGB1 siRNA and subsequently exposed to 10 μM GEF for 48 h to assess the expression of downstream pathways, with a particular focus on TLR4 and NF-κB. The mRNA and protein expressions of the relevant molecules were measured using the methods described previously. The results revealed that following HMGB1 downregulation by siRNA, GEF treatment could not increase HMGB1 and TLR4 mRNA expression anymore (Figures 2A,B). Moreover, the protein levels of HMGB1, TLR4 and NF-κB (p65 and p-p65) upregulation induced by GEF were not observed (Figures 2C,D). This finding demonstrates that HMGB1 plays a crucial role in mediating GEF-induced activation of TLR4 and NF-κB, suggesting that the activation of this signaling pathway is a key mechanism driving the hepatotoxic effects of GEF.

### 3.3 BBR alleviates GEF-induced hepatotoxicity in THLE-2/THLE-3 cells

To preliminarily explore the protective effect of BBR on GEF-induced liver injury *in vitro*, THLE-2 and THLE-3 cells were treated with GEF (10 μM) in combination with BBR at low, medium, and high concentrations (5 μM, 10 μM, and 20 μM, respectively) for 48 h. The levels of ALT, AST, and inflammatory cytokines (including TNF-α, IL-1β, and IL-6) in the cell supernatant were measured by ELISA, and cell apoptosis was assessed using the TUNEL assay.

**FIGURE 3:**
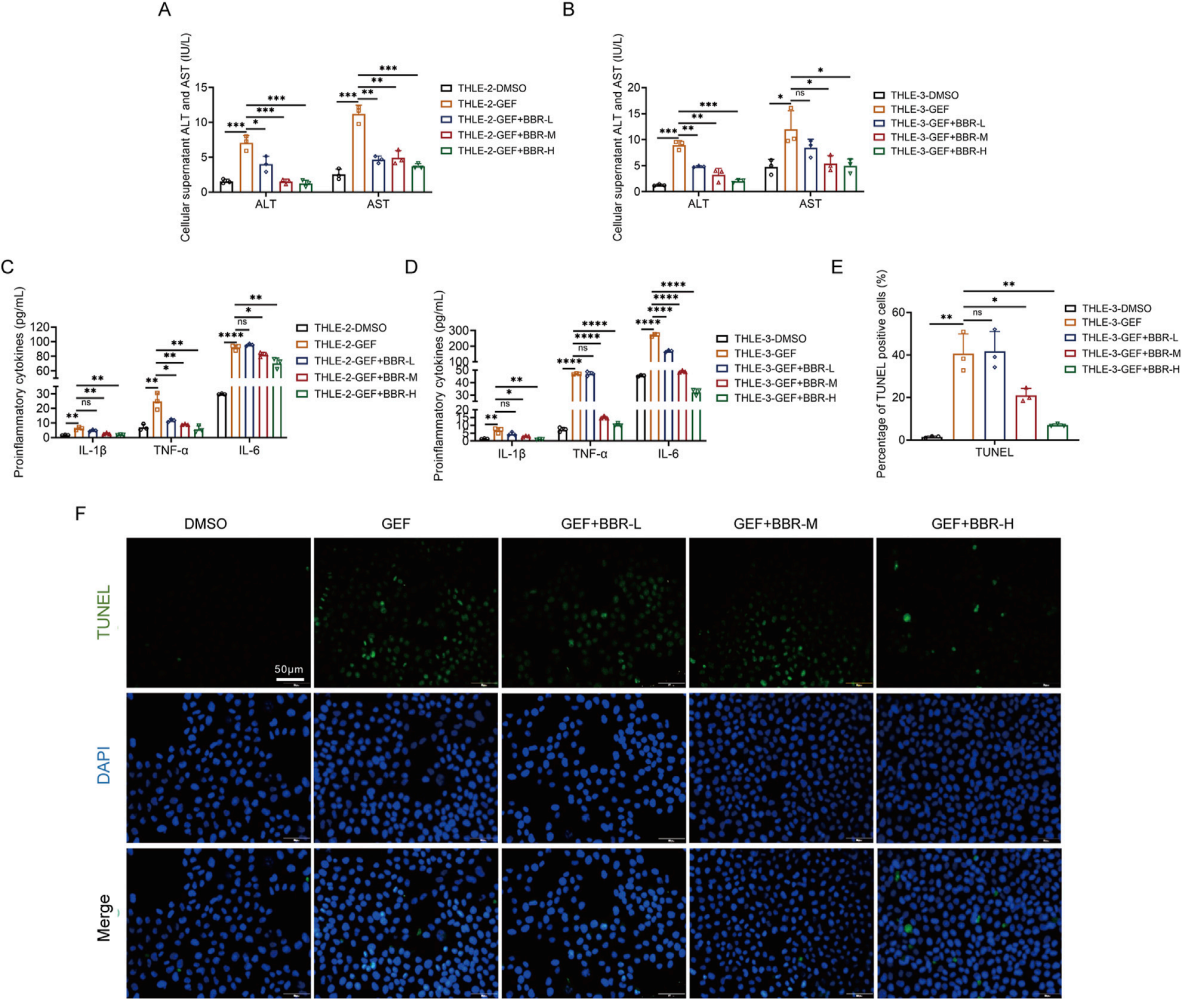
Berberine (BBR) attenuates gefitinib (GEF)-induced hepatotoxicity in THLE-2 and THLE-3 cells (n = 3). **(A,B)** Levels of ALT and AST in the cell supernatant. **(C,D)** Levels of inflammatory cytokine (TNF-α, IL-1β, and IL-6) in the cell supernatant. **(E)** Apoptosis measured by TUNEL assay (400×) in THLE-3 cells. **(F)** Representative images of apoptotic in THLE-3 cells. Cells were treated with 10 μM GEF in combination with low, medium, and high concentrations of BBR (5 μM, 10 μM, and 20 μM, denoted as BBR-L, BBR-M, and BBR-H, respectively) for 48 h. ns, no significance. *P < 0.05, **P < 0.01, ***P < 0.001, ****P < 0.0001. P-value < 0.05 was considered statistically significant.

The results demonstrated that co-treatment with BBR led to a dose-dependent decrease in the levels of ALT and AST in both cell lines (Figures 3A,B). Additionally, the levels of pro-inflammatory cytokines, including TNF-α, IL-1β, and IL-6 in the cell supernatants, were markedly reduced in a dose-dependent manner (Figures 3C,D). Higher concentrations of BBR leading to more significant reductions inflammatory responses, indicating that the protective effects of BBR are concentration-dependent. Furthermore, a significant reduction in cell apoptosis was observed with BBR treatment (Figures 3E,F; Supplementary Figures S1A,S1B), especially at high concentrations, which supports its role in mitigating GEF-induced hepatotoxicity.

### 3.4 BBR inhibits the HMGB1/TLR4/NF-κB pathway and reduces HMGB1 nuclear-to-cytoplasmic translocation in THLE-2/THLE-3 cells

**FIGURE 4:**
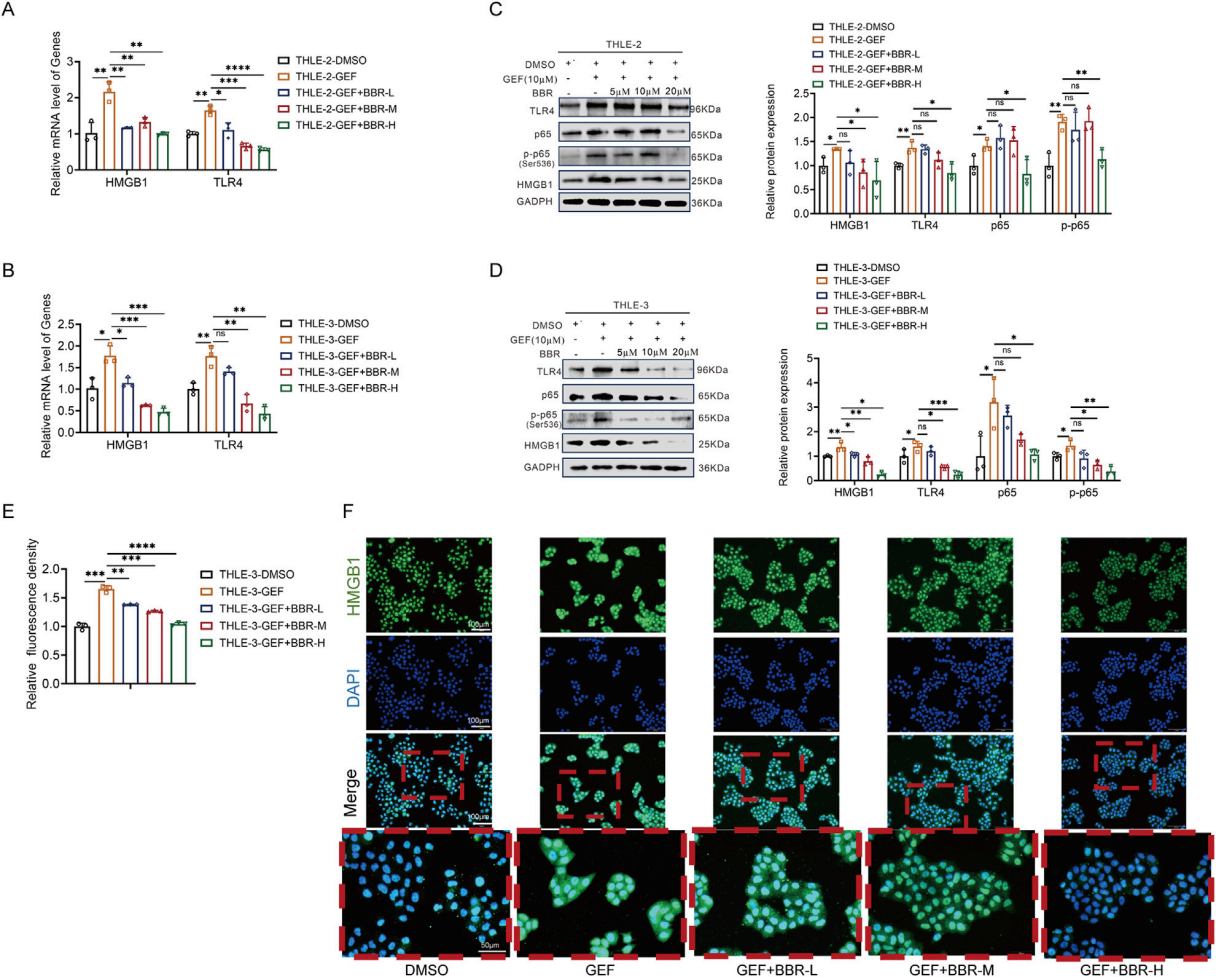
Berberine (BBR) inhibits the HMGB1/TLR4/NF-κB pathway and reduces HMGB1 nuclear-to-cytoplasmic translocation in THLE-2 and THLE-3 cells (n = 3). **(A,B)** mRNA expression of HMGB1 and TLR4. **(C,D)** Protein levels of HMGB1, TLR4, and NF-κB (p65 and p-p65). **(E)** Nuclear-to-cytoplasmic translocation of HMGB1 detected by immunofluorescence in THLE-3 cells. **(F)** Representative immunofluorescence images showing HMGB1 in the cytoplasm and nucleus of THLE-3 cells (200× and 400×). Cells were treated with 10 μM GEF and low, medium, and high concentrations of BBR (5 μM, 10 μM, and 20 μM, denoted as BBR-L, BBR-M, and BBR-H, respectively) for 48 h. ns, no significance. *P < 0.05, **P < 0.01, ***P < 0.001, ****P < 0.0001. P-value < 0.05 was considered statistically significant.

Next, the molecular mechanism of BBR’s protection against GEF-induced liver injury was investigated. Accordingly, THLE-2 and THLE-3 cells were co-treated with 20 μM gefitinib and BBR at concentrations of 5, 10, and 20 μM. The mRNA expression levels of HMGB1 and TLR4, as well as the protein levels of HMGB1, TLR4, and NF-κB, were analyzed using the methods described previously. Moreover, the nuclear-to-cytoplasmic translocation of HMGB1 was evaluated via immunofluorescence. The results showed that, compared to GEF treatment alone, co-treatment with BBR significantly downregulated the mRNA levels of HMGB1 and NF-κB (Figures 4A,B), as well as the protein expression of HMGB1, NF-κB, and TLR4 (Figures 4C,D). Furthermore, BBR significantly reduced the nuclear-to-cytoplasmic translocation of HMGB1 in both THLE-2 and THLE-3 cells, a key event associated with inflammation and liver injury (Figures 4E,F; Supplementary Figures S1C,S1D). Therefore, BBR alleviates GEF-induced hepatotoxicity by inhibiting the HMGB1/TLR4/NF-κB signaling pathway, reducing inflammation, and limiting the nuclear-to-cytoplasmic translocation of HMGB1.

### 3.5 BBR prevents GEF-induced hepatotoxicity and inhibits the HMGB1/TLR4/NF-κB pathway in rat

**FIGURE 5:**
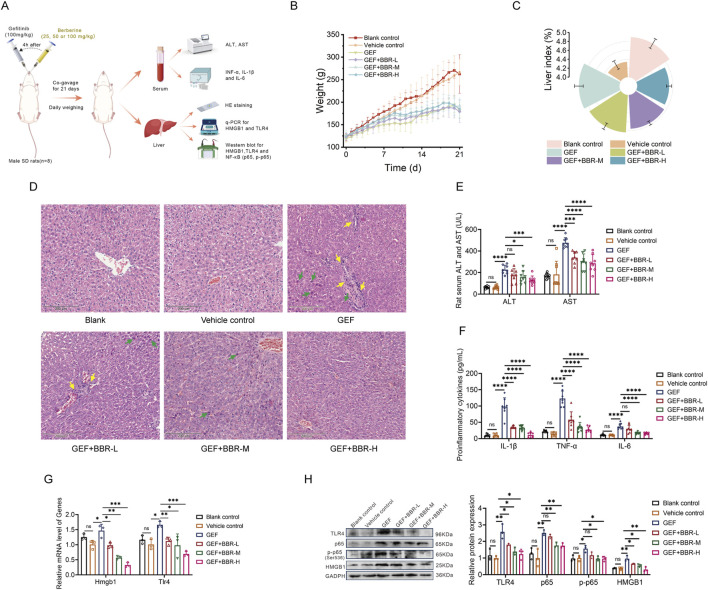
Berberine (BBR) prevents gefitinib (GEF)-induced hepatotoxicity and inhibits the HMGB1/TLR4/NF-κB pathway in rats (n = 8). **(A)** Animal experiment design. **(B)** Body weight changes during the treatment period. **(C)** Liver index (liver-to-body weight ratio). **(D)** Hepatic histopathological changes by HE staining (200×). Green arrows: eosinophilic change; yellow arrows: inflammatory cell infiltration. **(E)** Levels of ALT and AST in serum. **(F)** Levels of inflammatory cytokine (TNF-α, IL-1β, and IL-6) in serum. **(G)** mRNA expression of HMGB1 and TLR4 in liver. **(H)** Protein levels of HMGB1, TLR4, and NF-κB (p65 and p-p65) in liver. Rats were administered 100 mg/kg GEF for 3 weeks to establish a liver injury model, followed by co-administration of BBR at 25 mg/kg (BBR-L), 50 mg/kg (BBR-M), or 100 mg/kg (BBR-H) 4 h before daily GEF. ns, no significance. *P < 0.05, **P < 0.01, ***P < 0.001, ****P < 0.0001. P-value < 0.05 was considered statistically significant.

To further investigate the protective effect of BBR against GEF-induced liver injury *in vivo*, we established a rat model of GEF-induced liver injury by intragastric administration of 100 mg/kg GEF for 3 weeks. Following daily co-administration of BBR at low, medium, and high doses (25 mg/kg, 50 mg/kg, and 100 mg/kg, respectively), serum samples were collected for biochemical analysis of ALT and AST. Inflammatory markers, including TNF-α, IL-1β, and IL-6, were quantified using ELISA kits. Liver tissues were harvested for histopathological evaluation through HE staining. Additionally, mRNA expression levels of HMGB1 and TLR4, as well as protein levels of HMGB1, TLR4, and NF-κB, were measured as previously described (Figure 5A).

The results indicated that GEF treatment led to a reduction in body weight in rats (Figure 5B) and an increase in the liver index (liver-to-body weight ratio, Figure 5C). Pathological analysis through HE staining revealed hepatocellular injury, eosinophilic change, along with inflammatory cell infiltration (Figure 5D). Serum levels of transaminases (ALT, AST) and inflammatory cytokines (TNF-α, IL-1β, and IL-6) were also elevated (Figures 5E,F), indicating GEF-induced liver injury. These findings align with clinical reports of GEF-induced hepatotoxicity, which primarily manifests as hepatocellular injury (Zhou et al., 2024).

As depicted in Figures 5B–D, BBR co-treatment partially restored body weight and reduced the liver index and liver damage in a dose-dependent manner, with higher doses preserving liver architecture and reducing tissue injury more effectively. Serum ALT and AST levels were significantly lower in BBR-treated rats, with the greatest reductions observed at higher doses (Figure 5E). Furthermore, levels of pro-inflammatory cytokines, such as TNF-α, IL-1β, and IL-6, were reduced dose-dependently (Figure 5F), indicating that BBR mitigated the inflammatory response induced by GEF.

Mechanistically, BBR co-treatment effectively inhibited the activation of the HMGB1/TLR4/NF-κB pathway in rat liver. The mRNA and protein expression of HMGB1 and TLR4 was significantly reduced in the co-treated rats, with greater downregulation at higher doses (Figures 5G,H). Moreover, the protein expression of NF-κB was also significantly inhibited, particularly at the highest dose of BBR (Figure 5H). Consistent with *in vitro* data, BBR dose-dependently suppressed the HMGB1/TLR4/NF-κB axis, thereby attenuating liver inflammation and damage, and offering a promising therapeutic for GEF-induced hepatotoxicity.

## 4 Discussion

**FIGURE 6:**
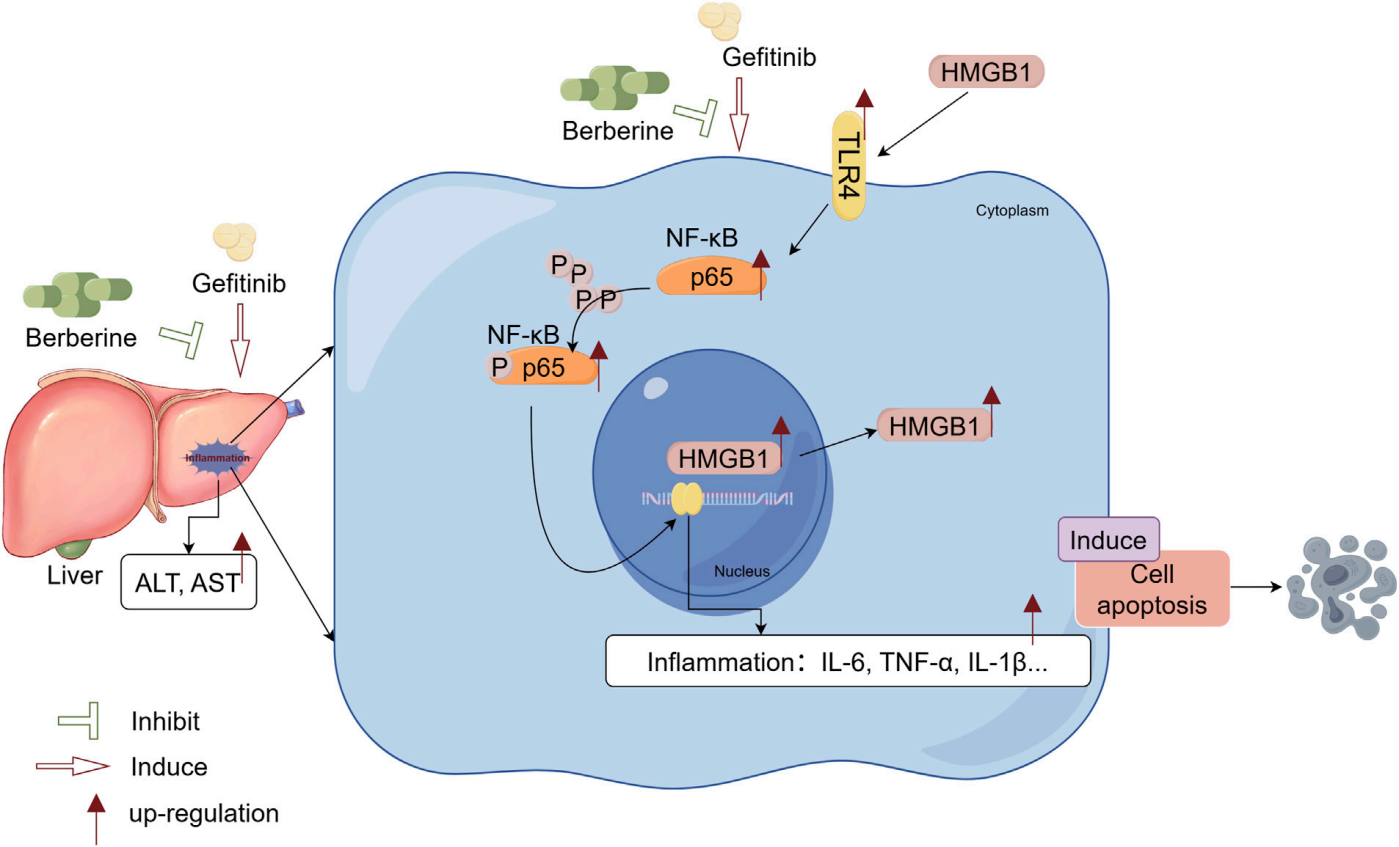
Berberine protects against gefitinib-induced liver injury by inhibiting the HMGB1/TLR4/NF-κB pathway. Berberine exerts dose-dependent hepatoprotective effects both *in vitro* and *in vivo* by suppressing HMGB1 expression and nuclear-to-cytoplasmic translocation, downregulating pro-inflammatory cytokines (IL-1β, IL-6, TNF-α), reducing serum aminotransferases (ALT, AST), and alleviating hepatocyte apoptosis.

Targeted therapies, especially the EGFR-TKI, have markedly improved outcomes in NSCLC, yet GEF-induced hepatotoxicity remains a critical clinical limitation. In the present study we demonstrate that GEF-induced liver injury is driven by sequential activation of the damage-associated molecular pattern HMGB1 and its downstream TLR4/NF-κB axis, and we reveal that, for the first time, pharmacological intervention with BBR dose-dependently attenuated liver injury, evidenced by downregulating HMGB1, inhibiting the TLR4/NF-κB signaling pathway, and suppressing the release of inflammatory mediators (Figure 6). Collectively, our findings establish the HMGB1/TLR4/NF-κB pathway as a central driver of gefitinib hepatotoxicity and identify BBR as a clinically relevant adjunct to prevent or mitigate GEF-associated liver injury.

DILI is a significant concern in the context of liver diseases, particularly during antitumor therapies (Chodup et al., 2025). DILI can result in serious outcomes, including treatment discontinuation, hepatic failure, and even death. Given that most small-molecule targeted agents undergo hepatic metabolism, DILI is a particularly common adverse effect in this drug class. Notably, GEF is associated with a significantly higher incidence of DILI compared to other agents in the same category, raising substantial concerns regarding its safety and clinical applicability. As a result, investigations into the mechanisms underlying GEF-induced liver toxicity have been more extensive than those for other EGFR-TKIs (Zhu et al., 2024). Developing effective strategies to prevent or alleviate GEF-induced liver injury is therefore essential to enhance patient adherence and optimize therapeutic outcomes in clinical practice.

Current research on the mechanisms of GEF-induced hepatotoxicity has identified multiple pathways that may contribute to liver injury. For instance, Li et al. (2009). Discovered that the active metabolites generated from the metabolism of gefitinib by CYP450 enzymes can induce oxidative stress damage. Zhang et al. (2021) found that GEF leads to abnormal expression of genes associated with the endoplasmic reticulum stress (ERS) pathway and apoptosis in a zebrafish model. Additionally, some studies have suggested that GEF-induced liver injury may be related to autophagy (Luo et al., 2021). Moreover, GEF can activate cells to release HMGB1, which in turn triggers immune responses and immune-related adverse events and induces cell apoptosis. Research has confirmed that HMGB1 is associated with impaired mitochondrial oxidative phosphorylation and disrupted free fatty acid (FFA) β-oxidation related to enhanced ER stress (Lin et al., 2020). When cells are under stress or tissues are injured, HMGB1 can undergo post-translational modifications such as acetylation, phosphorylation, methylation, or oxidation. Additionally, HMGB1 can regulate autophagy and apoptosis (Khambu et al., 2019). Overall, oxidative stress, endoplasmic reticulum stress, apoptosis, and autophagy appear to interact intricately with HMGB1 in the pathogenesis of GEF-induced liver injury.

Based on previous research, we hypothesized that HMGB1 plays a pivotal role in GEF-induced liver injury. Our results support the hypothesis, and align with previous findings by Noguchi et al. (2021) and Zhang et al. (2015). Furthermore, the specific binding ligands and downstream factors of HMGB1 involved in GEF-induced liver injury have not been thoroughly investigated before. In this study, we demonstrate for the first time that the activation of the HMGB1/TLR4/NF-κB signaling pathway triggers the release of inflammatory factors and induces apoptosis, which is a key mechanism underlying GEF-induced liver injury. This pathway is a critical contributor to GEF-induced liver injury and represents a promising therapeutic target for clinical intervention.

At present, current clinical guidelines for managing DILI caused by EGFR-TKIs like GEF primarily emphasize reactive management strategies, including discontinuation of suspected hepatotoxic medications, switching to alternative targeted therapies, and administration of appropriate hepatoprotective agents. In cases of acute severe liver failure, liver transplantation is necessary. Regarding proactive prevention of gefitinib-associated DILI risk, comprehensive monitoring and risk stratification are recommended, while evidence-based pharmacological prophylaxis is currently lacking. Our study identifies BBR as a potential prophylactic agent that could be used to reduce GEF-related hepatotoxicity and improve treatment continuity.

Among potential hepatoprotective agents, traditional Chinese medicine (TCM), specifically the natural alkaloid BBR, has gained attention due to its multimodal mechanisms and favorable safety profile. For example, BBR can modulate lipid metabolism, regulate bile acids, and exert anti-inflammatory, antioxidant, and anti-fibrotic effects in the treatment of liver diseases (Bansod et al., 2021). Indeed, extensive research has demonstrated BBR’s protective effects against liver injury induced by various agents, including CCl4 (Han et al., 2019), cyclophosphamide (Germoush and Mahmoud, 2014), paraquat (Javad-Mousavi et al., 2016), acetaminophen (Zhao et al., 2018), doxorubicin (Chen et al., 2016), methotrexate (Mahmoud et al., 2017), and others. Critically, its role in mitigating GEF-specific hepatotoxicity had not been investigated prior to our work. Our findings provide mechanistic evidence supporting BBR’s potential as an adjunctive hepatoprotective strategy in gefitinib-treated patients.

Research has shown that BBR can lower HMGB1, inhibit the HMGB1/NF-κB pathway, and decrease pro-inflammatory cytokine production and reduce inflammatory damage, including myocardial injury (Goto et al., 2024; Long et al., 2023), liver injury (Gendy et al., 2022), thrombosis (Wei et al., 2022), cerebral injury (Tentu et al., 2025), renal injury (Yang et al., 2024), and DILI (Zhao et al., 2018). In this study, we demonstrated that BBR could reverse GEF-induced activation of the HMGB1/TLR4/NF-κB pathway by downregulating its expression. This led to a decrease in the levels of ALT, AST, and inflammatory factors in cell supernatants and rat serum, as well as reduced cell apoptosis. Furthermore, HMGB1’s functional versatility is highlighted by its distinct roles inside and outside the cell. Intracellularly, HMGB1 is crucial for DNA maintenance, gene regulation, and the initiation of various cellular processes, while extracellular HMGB1 functions as a damage-associated molecular pattern (DAMP), triggering immune responses to alert the host to cellular damage (Khambu et al., 2019). Our study also found that BBR reduced the nuclear-to-cytoplasmic translocation of HMGB1, suggesting that it may decrease the proportion of HMGB1 released extracellularly, thereby reducing the activation of the damage-associated cascade triggered by HMGB1 as a DAMP molecule.

In summary, our findings confirm that BBR directly inhibits HMGB1 expression and its downstream signaling, particularly the TLR4/NF-κB pathway. This inhibition demonstrates the therapeutic value of BBR against GEF-induced liver injury and provides new insights into preventive strategies for managing GEF-associated hepatotoxicity. Our results underscore the translational potential of BBR as an adjunctive agent in clinical practice, particularly for NSCLC patients undergoing EGFR-TKI therapy.

This study has several limitations. First, although we elucidated the HMGB1/TLR4/NF-κB axis as a key pathway in GEF-induced hepatotoxicity, the multifactorial nature of DILI suggests involvement of additional mechanisms. Notably, recent evidence implicates autophagy dysregulation and endoplasmic reticulum (ER) stress in GEF-induced hepatotoxicity, which may crosstalk with HMGB1 signaling (Luo et al., 2021; Guan et al., 2022). Our study did not assess HMGB1 subcellular localization or hepatocyte apoptosis in liver tissues, nor did we examine autophagy-related responses *in vivo*. These limitations restrict our understanding of the full mechanistic landscape. These gaps preclude a comprehensive mechanistic understanding. To address this, we are currently conducting extended investigations evaluating HMGB1 trafficking, apoptotic cascades, and autophagic responses in preclinical models, with findings to be reported separately. Second, while we demonstrated BBR’s anti-inflammatory efficacy, its multiple mechanisms such as antioxidant, autophagy modulation, regulation of gut microbiota metabolites, remains insufficiently investigated. Additionally, BBR’s low oral bioavailability (<5%) necessitates high-dose gavage administration, which differs significantly from clinical administration dosage. Future studies should address these limitations to better understand berberine’s therapeutic potential in clinical settings.

## 5 Conclusion

Our study reveals that activation of the HMGB1/TLR4/NF-κB signaling pathway is a critical mechanism in GEF-induced liver injury. Berberine exerts significant hepatoprotective effects by downregulating HMGB1 and suppressing this inflammatory cascade. These findings suggest that BBR may serve as an effective strategy to prevent gefitinib-induced hepatotoxicity and provide novel mechanistic insights into its pathogenesis.

## 6 Summary

Our study shows that berberine (BBR) protects against gefitinib-induced liver injury by inhibiting the HMGB1/TLR4/NF-κB signaling pathway. *In vitro*, BBR reduced transaminases, inflammatory cytokines, and HMGB1 translocation induced by gefitinib. *In vivo*, BBR improved liver function, lowered transaminase levels, and decreased tissue damage dose-dependently. It also suppressed HMGB1, TLR4, and NF-κB expression at both mRNA and protein levels. These results suggest BBR as a potential strategy to protect patients from gefitinib-induced liver toxicity during non-small cell lung cancer treatment.

## Data Availability

The original contributions presented in the study are included in the article/Supplementary Material, further inquiries can be directed to the corresponding authors.
