# Supplementary material for: Berberine protects against gefitinib-induced liver injury by inhibiting the HMGB1/TLR4/NF-κB pathway
Source: Front Pharmacol. 2025 Aug 26;16:1645634. doi: 10.3389/fphar.2025.1645634 (PMC12417729; doi:10.3389/fphar.2025.1645634)
Supplement: Supplementary file 1 [file Table1.docx]

Table S1 Basic information of antibodies used in the study

| Antibodies | Manufacturer | Lot number | Species | Dilution |
| --- | --- | --- | --- | --- |
| GAPDH | Proteintech | #81640-5-RR | Rabbit | 1:5000 |
| HMGB1 | Proteintech | #10829-1-AP | Rabbit | 1: 5000 |
| TLR4 | Proteintech | #30400-1-AP | Rabbit | 1:1000 |
| NF-κB p65 | Affinity | #AF5006 | Rabbit | 1:1000 |
| Phospho-NF-κB p65 (Ser536) | Affinity | #AF2006 | Rabbit | 1:1000 |
| HRP-Goat Anti-Rabbit IgG (H+L) | ZSGB-BIO | # ZB-2301 | Goat | 1:5000 |
| Goat-Anti-Rabbit IgG/Alexa Fluor 488 | ZSGB-BIO | #ZF-0511 | Goat | 1:50 |
