# Supplementary figures and images for: Berberine protects against gefitinib-induced liver injury by inhibiting the HMGB1/TLR4/NF-κB pathway
Source: Front Pharmacol. 2025 Aug 26;16:1645634. doi: 10.3389/fphar.2025.1645634 (PMC12417729; doi:10.3389/fphar.2025.1645634)

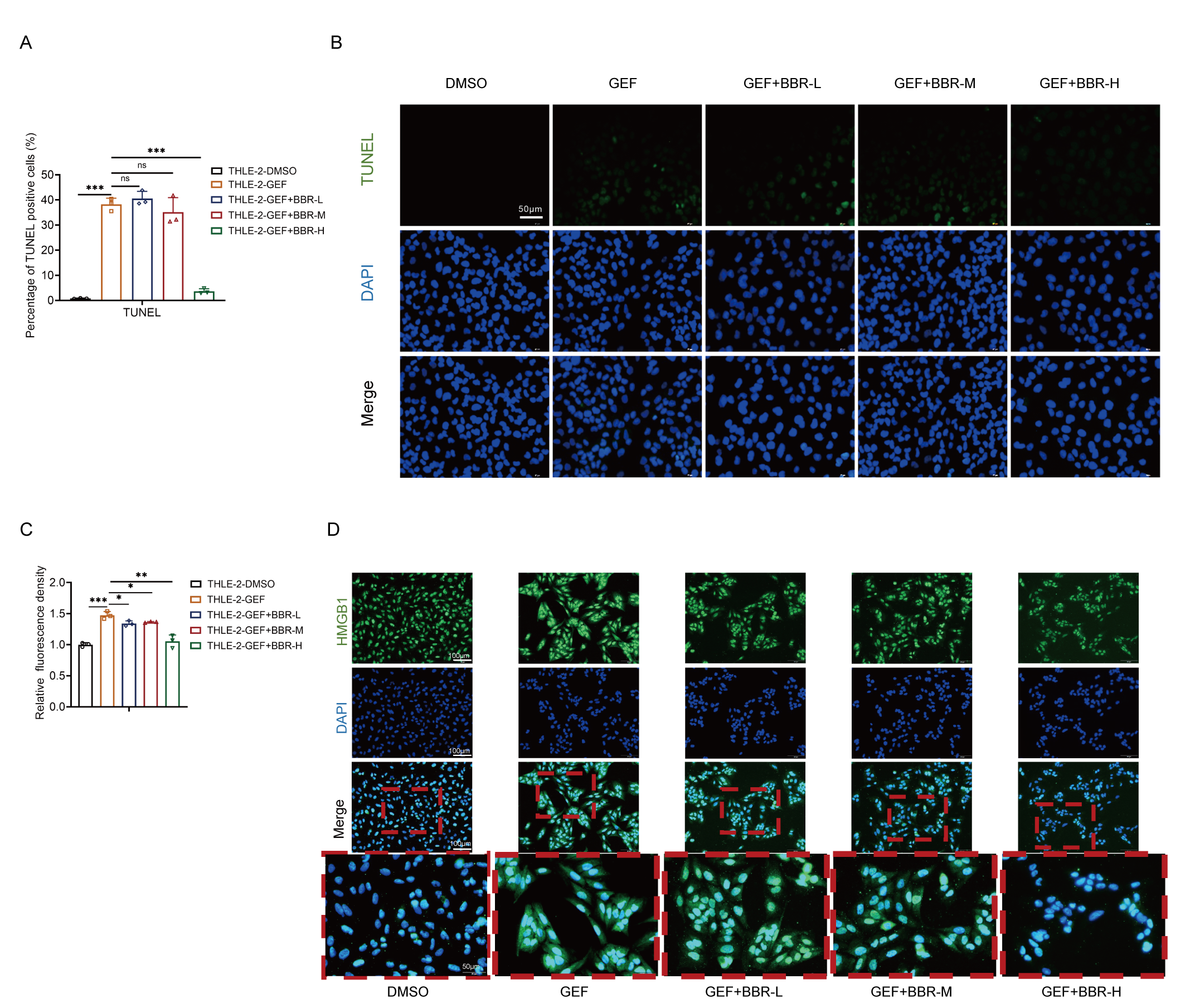

Supplement: Supplementary file 2 [file Image1.tif]
